# Timing of dialysis in acute kidney injury using routinely collected data and dynamic treatment regimes

**DOI:** 10.1186/s13054-022-04252-1

**Published:** 2022-11-28

**Authors:** Paweł Morzywołek, Johan Steen, Stijn Vansteelandt, Johan Decruyenaere, Sigrid Sterckx, Wim Van Biesen

**Affiliations:** 1 Department of Applied Mathematics, Computer Science and Statistics, Ghent University, Ghent, Belgium; 2 Centre for Justifiable Digital Healthcare, Ghent University Hospital, Ghent, Belgium; 3 Renal Department, Ghent University Hospital 0K12IA, Corneel Heymanslaan 10, 9000 Ghent, Belgium; 4 Department of Medical Statistics, London School of Hygiene and Tropical Medicine, London, UK; 5 Department of Intensive Care Medicine, Ghent University Hospital, Ghent, Belgium; 6 Bioethics Institute Ghent, Department of Philosophy and Moral Sciences, Ghent University, Ghent, Belgium

## Abstract

**Background and objectives:**

Defining the optimal moment to start renal replacement therapy (RRT) in acute kidney injury (AKI) remains challenging. Multiple randomized controlled trials (RCTs) addressed this question whilst using absolute criteria such as pH or serum potassium. However, there is a need for identification of the most optimal cut-offs of these criteria. We conducted a causal analysis on routinely collected data (RCD) to compare the impact of different pre-specified dynamic treatment regimes (DTRs) for RRT initiation based on time-updated levels of potassium, pH, and urinary output on 30-day ICU mortality.

**Design, setting, participants, and measurements:**

Patients in the ICU of Ghent University Hospital were included at the time they met KDIGO-AKI-stage ≥ 2. We applied inverse-probability-of-censoring-weighted Aalen–Johansen estimators to evaluate 30-day survival under 81 DTRs prescribing RRT initiation under different thresholds of potassium, pH, or persisting oliguria.

**Results:**

Out of 13,403 eligible patients (60.8 ± 16.8 years, SOFA 7.0 ± 4.1), 5622 (63.4 ± 15.3 years, SOFA 8.2 ± 4.2) met KDIGO-AKI-stage ≥ 2. The DTR that delayed RRT until potassium ≥ 7 mmol/l, persisting oliguria for 24–36 h, and/or pH < 7.0 (non-oliguric) or < 7.2 (oliguric) despite maximal conservative treatment resulted in a reduced 30-day ICU mortality (from 12.7% [95% CI 11.9–13.6%] under current standard of care to 10.5% [95% CI 9.5–11.7%]; risk difference 2.2% [95% CI 1.3–3.8%]) with no increase in patients starting RRT (from 471 [95% CI 430–511] to 475 [95% CI 342–572]). The fivefold cross-validation benchmark for the optimal DTR resulted in 30-day ICU mortality of 10.7%.

**Conclusions:**

Our causal analysis of RCD to compare RRT initiation at different thresholds of refractory low pH, high potassium, and persisting oliguria identified a DTR that resulted in a decrease in 30-day ICU mortality without increase in number of RRTs. Our results suggest that the current criteria to start RRT as implemented in most RCTs may be suboptimal. However, as our analysis is hypothesis generating, this optimal DTR should ideally be validated in a multicentric RCT.

**Supplementary Information:**

The online version contains supplementary material available at 10.1186/s13054-022-04252-1.

## Introduction

Acute kidney injury (AKI) is a prevalent condition with a substantial morbidity and mortality [1, 2]. Whereas renal replacement therapy (RRT) can be life-saving in this setting, it also brings potentially life-threatening complications [3], such as hypotension, bleeding or infection, and a huge logistical and financial burden [4]. In addition, a substantial number of patients with AKI recover spontaneously without need for RRT [5]. Starting RRT too early or in patients without good indication can therefore shift the cost–benefit of RRT to the negative side. Defining the optimal decision strategy for initiating RRT in a way that optimally balances benefits and drawbacks remains challenging. Several randomized controlled trials (RCTs) have tried to develop insight [3, 5–9]. An individual patient data meta-analysis [5] of these RCTs found no evidence that early start of RRT would have a beneficial impact on ICU survival. Furthermore, while delayed strategies may avoid RRT in a substantial number of patients [5], evidence suggests it may cause harm [6]. Lack of clarity thus remains on when to best initiate RRT. This is partly because RCTs have so far only evaluated very specific treatment rules, so-called dynamic treatment regimes (DTRs), which require immediately starting RRT once specific absolute criteria (defined as refractory metabolic acidosis (low pH), hyperkalaemia, and/or intractable volume overload [10] are attained. However, current guidelines do not quote specific thresholds for these absolute criteria [10–13].

Taking these considerations into account, the question when to initiate RRT for patients with AKI has thus only been answered partially. There is in particular a need to identify optimal cut-offs for the above-mentioned absolute criteria. Traditional RCTs are not feasible for this purpose as many combinations of thresholds for these criteria are possible, even if restricted to the most important ones such as potassium, pH, and volume overload. In view of this, the main goal of this study is to use routinely collected data (RCD) to investigate whether pre-specified DTRs for initiation of RRT based on combinations of different thresholds of pH, potassium, and persistent oliguria can improve 30-day survival in ICU patients who fulfilled KDIGO-AKI stage ≥ 2 criteria as compared to the current standard of care strategy. We will address this question by applying target trial emulation framework (cloning–censoring–weighting approach) to RCD. Such a target trial emulation framework typically intends to generate hypotheses on the outcome of different treatment scenarios, the most optimal of which should then later be validated in an RCT [14].

Drawing evidence from RCD faces many challenges, however. Whereas adjustment for confounding is essential, it is also challenging as key confounders (e.g. evolving disease severity, as captured by daily SOFA scores) not only influence the outcome of interest, but also later treatment decisions, and are at the same time themselves affected by earlier treatment decisions (time-varying confounding). This makes it difficult to adjust for confounding in a way that prevents overadjustment, a problem often present with techniques such as propensity score matching. To accommodate this, we will address our comparative effectiveness question within a target trial emulation framework for causal analysis of RCD. This framework revolves around specifying the hypothetical randomized trial one would ideally conduct to answer the comparative effectiveness question of interest [14–17]. This, in turn, clarifies the research question at hand and provides a roadmap for emulating that “target” trial from observational RCD [14, 18]. Such a roadmap permits to appropriately tackle time-varying confounding, eliminate other easily avoidable (but common) sources of bias [19, 20] and to generate hypotheses about optimal treatment strategies, which could then later be validated in a prospective RCT [14, 21]. To be more specific, we will make use of a cloning–censoring–weighting approach for estimation and evaluation of 30-day ICU mortality under different pre-specified DTRs. Inverse-probability-of-censoring-weighting [22] will be applied to adjust for confounding by time-varying covariates. Such techniques have been successfully used, e.g. to optimize the time of initiating antiretroviral therapy in patients with Human Immunodeficiency Virus (HIV) [23]. By thus applying these techniques to RCD, one can evaluate 30-day ICU mortality under a range of clinically relevant DTRs [14]. The most promising DTRs may then later be validated in a traditional RCT.

## Materials and methods

### Data source

The ICU of Ghent University Hospital has 52 beds, all equipped with an Intensive Care Information System (ICIS) (Centricity Critical Care, GE Healthcare, Germany). Longitudinal patient data from monitors, ventilators, pumps, radiology, and laboratory results and administered medication are uploaded in real time into the system.

### Study population

Our analysis was restricted to adult patients (> 18 years old), admitted to the surgical, post-cardiac surgical or medical ICU between January 1, 2013, and December 31, 2017, and who developed KDIGO-AKI stage ≥ 2, operationalized according to a broad definition as described elsewhere [24]. Patients were excluded when they met one of the following criteria: recorded RRT history; missing baseline weight measurement; or predefined registered Do Not Resuscitate (DNR) restrictions to start RRT.

RRT in the ICU was provided at the discretion of the treating nephrologist and intensivist as either intermittent haemodialysis (IHD), slow low-efficient daily dialysis (SLEDD), or continuous renal replacement therapy (CRRT).

The study was approved by the Ethics Committee of the University Hospital Ghent (EC nr 201-0705).

Because of the highly technical nature of our analysis, patient and public involvement was not considered in the planning and conduct of our project. We do plan dissemination of the results, as these might be relevant in the shared decision making of initiation of renal replacement therapy.

### Covariates

The raw data relevant for our study were extracted in an anonymized way from the ICIS database. The baseline covariates included age on admission, weight, gender, ICU admission time, admission category, and pre-existing underlying chronic kidney disease. Time-varying covariates included laboratory values (potassium, urea, magnesium, creatinine, and arterial pH), cumulative fluid intake and output, FiO_2_, SpO_2_, P/F ratio, PaO_2_, DNR code, and daily updated SOFA subscores (see Additional file 1: Appendix for a more detailed overview of the covariates used in the analysis).

### Treatment strategies

We compared different hypothetical treatment strategies that would initiate RRT, when the KDIGO-AKI stage ≥ 2 creatinine criterion was met and when in the last 24 h at least one of the following events occurred: pH fell below a particular threshold value (values of 7.0, 7.1, and 7.2), potassium level exceeded a particular threshold value (for threshold values of 6.0, 6.5, and 7.0 mEq/l), or oliguric KDIGO-AKI stage 3 (defined as urinary output < 0.3 ml/kg/h for 24 h) was reached. The latter was used as a surrogate for persisting or worsening oligo-anuria during 24–36 h. Under the considered DTR, the decision about RRT initiation is performed every 24 h starting from the day of inclusion based on the information available up to a given decision point. In our ICU, there is a wide array of standardized operating protocols for non-RRT management of patients with hyperkalaemia, metabolic acidosis, or oliguria. The threshold values are thus to be considered as values in patients refractory to these conservative treatments.

**Fig. 1:**
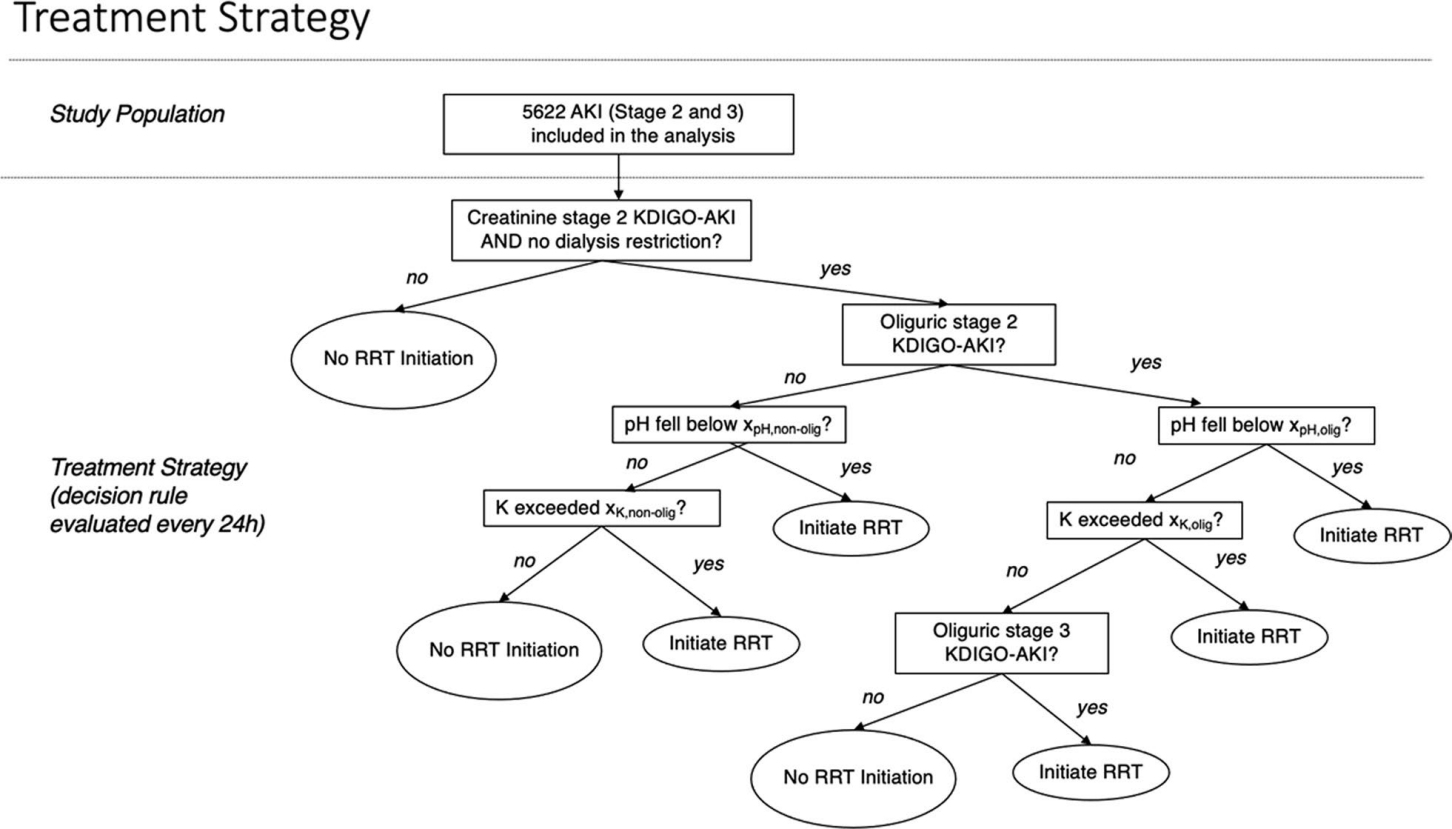
Treatment strategy flowchart

As there was heterogeneity between patients depending on whether they had or had not reached the oliguric (vs only the creatinine) criterion for KDIGO-AKI stage ≥ 2, we evaluated treatment regimes that considered separate thresholds for K and/or pH depending on whether or not oliguric KDIGO-AKI stage 2 had been met by the time of considering initiating RRT (Fig. 1). This resulted finally in the comparison of 81 different dynamic treatment regimes (Table 1).

**Table 1:** List of thresholds in the considered treatment strategies

	*X*_pH, olig_	*X*_pH, non-olig_	*X*_K, olig_	*X*_K, non-olig_
1	7.0	7.0	6.0	6.0
2	7.0	7.0	6.5	6.0
3	7.0	7.0	7.0	6.0
4	7.0	7.0	6.0	6.5
5	7.0	7.0	6.5	6.5
6	7.0	7.0	7.0	6.5
7	7.0	7.0	6.0	7.0
8	7.0	7.0	6.5	7.0
9	7.0	7.0	7.0	7.0
10	7.1	7.0	6.0	6.0
11	7.1	7.0	6.5	6.0
12	7.1	7.0	7.0	6.0
13	7.1	7.0	6.0	6.5
14	7.1	7.0	6.5	6.5
15	7.1	7.0	7.0	6.5
16	7.1	7.0	6.0	7.0
17	7.1	7.0	6.5	7.0
18	7.1	7.0	7.0	7.0
19	7.2	7.0	6.0	6.0
20	7.2	7.0	6.5	6.0
21	7.2	7.0	7.0	6.0
22	7.2	7.0	6.0	6.5
23	7.2	7.0	6.5	6.5
24	7.2	7.0	7.0	6.5
25	7.2	7.0	6.0	7.0
26	7.2	7.0	6.5	7.0
27	7.2	7.0	7.0	7.0
28	7.0	7.1	6.0	6.0
29	7.0	7.1	6.5	6.0
30	7.0	7.1	7.0	6.0
31	7.0	7.1	6.0	6.5
32	7.0	7.1	6.5	6.5
33	7.0	7.1	7.0	6.5
34	7.0	7.1	6.0	7.0
35	7.0	7.1	6.5	7.0
36	7.0	7.1	7.0	7.0
37	7.1	7.1	6.0	6.0
38	7.1	7.1	6.5	6.0
39	7.1	7.1	7.0	6.0
40	7.1	7.1	6.0	6.5
41	7.1	7.1	6.5	6.5
42	7.1	7.1	7.0	6.5
43	7.1	7.1	6.0	7.0
44	7.1	7.1	6.5	7.0
45	7.1	7.1	7.0	7.0
46	7.2	7.1	6.0	6.0
47	7.2	7.1	6.5	6.0
48	7.2	7.1	7.0	6.0
49	7.2	7.1	6.0	6.5
50	7.2	7.1	6.5	6.5
51	7.2	7.1	7.0	6.5
52	7.2	7.1	6.0	7.0
53	7.2	7.1	6.5	7.0
54	7.2	7.1	7.0	7.0
55	7.0	7.2	6.0	6.0
56	7.0	7.2	6.5	6.0
57	7.0	7.2	7.0	6.0
58	7.0	7.2	6.0	6.5
59	7.0	7.2	6.5	6.5
60	7.0	7.2	7.0	6.5
61	7.0	7.2	6.0	7.0
62	7.0	7.2	6.5	7.0
63	7.0	7.2	7.0	7.0
64	7.1	7.2	6.0	6.0
65	7.1	7.2	6.5	6.0
66	7.1	7.2	7.0	6.0
67	7.1	7.2	6.0	6.5
68	7.1	7.2	6.5	6.5
69	7.1	7.2	7.0	6.5
70	7.1	7.2	6.0	7.0
71	7.1	7.2	6.5	7.0
72	7.1	7.2	7.0	7.0
73	7.2	7.2	6.0	6.0
74	7.2	7.2	6.5	6.0
75	7.2	7.2	7.0	6.0
76	7.2	7.2	6.0	6.5
77	7.2	7.2	6.5	6.5
78	7.2	7.2	7.0	6.5
79	7.2	7.2	6.0	7.0
80	7.2	7.2	6.5	7.0
81	7.2	7.2	7.0	7.0

Each considered treatment strategy is depicted through the treatment strategy flowchart (Fig. 1) and is unique through a combination of four thresholds: “xpH,olig” (pH threshold for patients that had met oliguric KDIGO-AKI stage 2 condition at the time of considering initiating RRT), “xpH,non-olig” (pH threshold for patients that had not met oliguric KDIGO-AKI stage 2 condition at the time of considering initiating RRT), “xK,olig” (serum potassium threshold for patients that had met oliguric KDIGO-AKI stage 2 condition at the time of considering initiating RRT), and “xK,non-olig” (serum potassium threshold for patients that had not met oliguric KDIGO-AKI stage 2 condition at the time of considering initiating RRT)

### Study outcome

The primary outcome was ICU mortality over the 30-day follow-up period under considered DTR starting from the moment the criteria for KDIGO-AKI stage ≥ 2 were reached. Patients were followed until the first of the following events: death; ICU discharge; or day 30 of follow-up. The secondary outcome was the number of patients starting RRT under the considered DTRs.

### Statistical analysis

To evaluate the performance of different DTRs using RCD, we applied a three-step estimation procedure referred to as “cloning, censoring, and weighting” [14, 21, 25, 26]. In the first step (cloning step), we create an extended data set with 81 copies of each patient, i.e. one “clone” for each DTR that we wish to evaluate. We then follow each of the 81 clones of each individual over time. If the treatment initiation decision based on the pre-specified but hypothetical DTR the clone was assigned to, did not coincide with the observed treatment for that patient, then the clone was censored for this DTR from this timepoint onward (censoring step). This artificial censoring may select a patient population over time that is no longer comparable to the full patient population in terms of time-varying prognostic factors for the outcome due to the problem of time-varying confounding. This can be remedied by inverse-probability-of-censoring (IPC) weighting (weighting step). The main idea behind IPC weighting is to re-construct the original population as if there was no censoring by assigning more weight to uncensored patients (or their clones) who are more likely to get censored and are hence underrepresented under the considered DTR. The IPC weights are equal to 1 over the probability of remaining uncensored under the considered DTR up to considered timepoint. We estimated this probability using a pooled logistic regression model including main effects of the baseline and time-varying covariates and time mentioned in the section “Covariates”. Given the obtained IPC weights, we estimated the cumulative incidence of the event of interest (i.e. percentage of patients who have died in the ICU by the considered time) under each DTR, over the considered 30-day period (taking into account the competing event, ICU discharge), using the IPC-weighted Aalen–Johansen estimator [22, 26, 27]. Confidence intervals were calculated using the infinitesimal jackknife variance estimator (using R package “survival”). As secondary outcome, the expected number of patients initiated on RRT based on the considered DTRs was estimated [26], and confidence intervals were obtained using the non-parametric bootstrap based on 500 resamples. The statistical analysis is described in more detail in Additional file 1. All analyses were conducted in R (version 4.0.1).

### Internal validation

In our analysis, we choose the best-performing DTR and report the resulting 30-day ICU mortality on the full available dataset. This may lead to a degree of overoptimism about the reported optimal performance as compared to an evaluation on an independent test set. Therefore, to assess the level of overoptimism in our analysis we computed a fivefold cross-validation benchmark for the optimal DTR (see Morzywolek et al. [26] for a detailed description of the methodology). The purpose of cross-validation is to assess the performance of the best-performing DTR on a separate dataset than the one used to choose it.

## Results

Of the complete data set of 13,403 eligible patients (62.2% male, 60.8 ± 16.8 years of age, SOFA 7.0 ± 4.1), 5622 (65.3% male, 63.4 ± 15.3 years of age, SOFA 8.2 ± 4.2) met our in- and exclusion criteria (Table 2).

**Table 2:** Demographic data of the cohort

	Complete cohort	KDIGO-AKI stage ≥ 2 cohort	KDIGO-AKI stage ≥ 2 cohort that received RRT (within 30-day follow-up)	KDIGO-AKI stage ≥ 2 cohort that did not receive RRT (within 30-day follow-up)
Number of patients	13,403	5622	471	5151
Age	60.8 ± 16.8	63.4 ± 15.3	64.0 ± 15.1	63.3 ± 15.4
Gender	62.2% male	65.3% male	62.2% male	65.5% male
SOFA score (at ICU admission)	7.0 ± 4.1	8.2 ± 4.2	11.4 ± 4.4	7.9 ± 4.1
Ventilated	91.4%	92.0%	95.1%	91.7%
Number of days of stay at ICU before AKI stage 2	–	1.05	0.66	1.09
% CKD*	16.3%	–	–	–
% Mortality before 30-day ICU^£^	–	12.7%	43.3%	9.9%

^£^In the study, we consider 30-day ICU mortality counting from the time of AKI (stage 2) diagnosis; therefore, it is not defined for non-AKI cohort

*Only mentioned in the overall cohort as the other groups do not contain patients with pre-existing CKD, as this is an exclusion criterion that we apply to define our population of interest

**Fig. 2:**
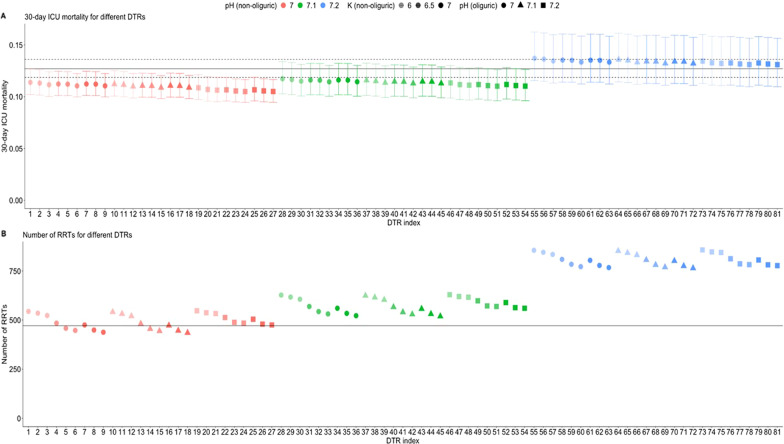
30-day ICU mortality with 95% confidence intervals and number of patients started on renal replacement therapy under the different regimes. Indices in the *X*-axis refer to the different DTRs as described in Table 1. Full horizontal line represents mortality (upper panel) and number of patients starting RRT under the current standard of care. Dashed lines represent 95% confidence intervals

Figure 2 depicts the estimated 30-day ICU mortality (upper panel) and number of patients starting RRT (lower panel) under the current standard of care (reference) and under the different considered DTRs. Additional file 1: Appendix Fig. S1 offers a more refined presentation of the results.

**Fig. 3:**
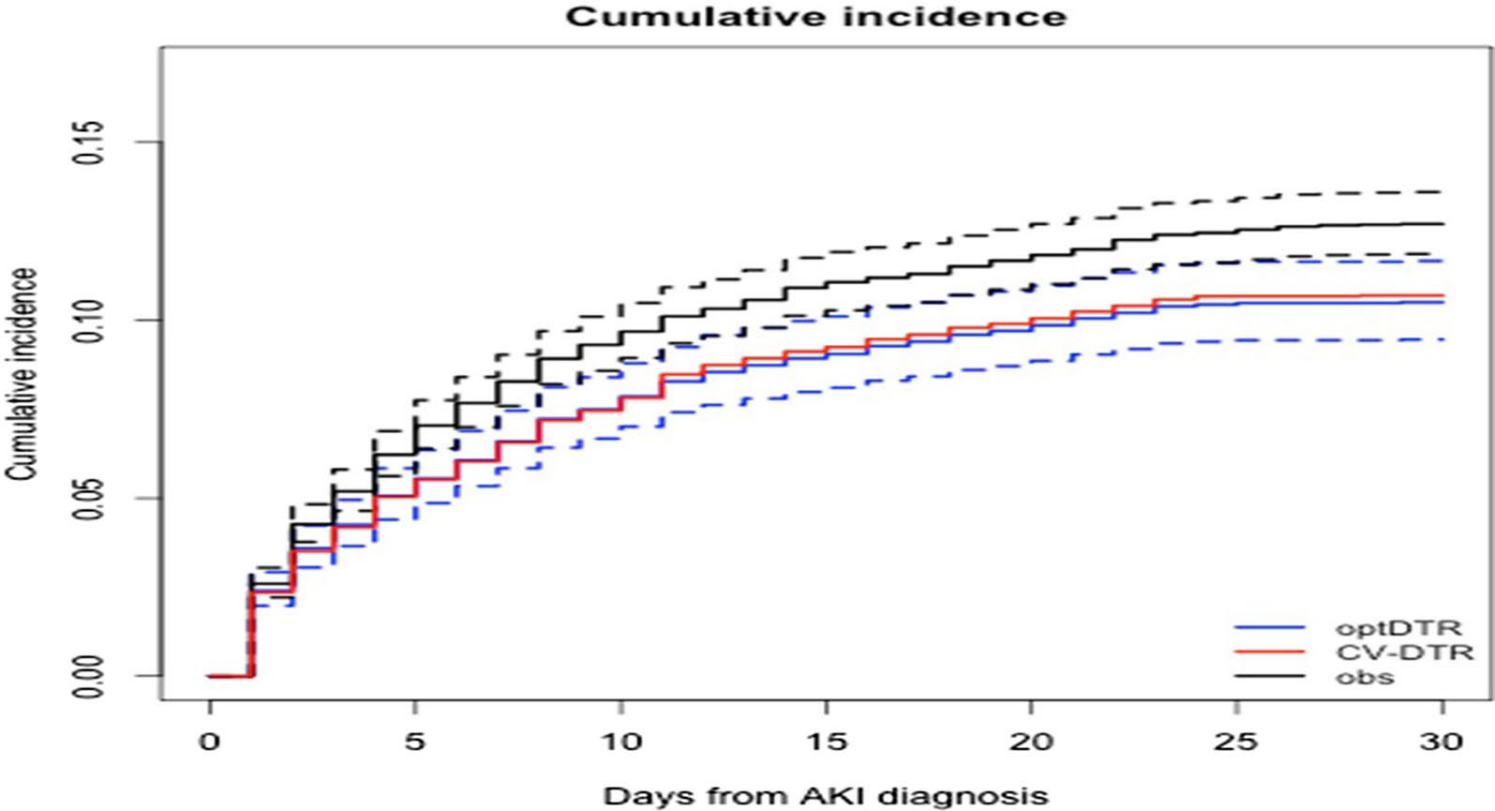
Cumulative incidence of 30-day ICU mortality. Curves show ICU mortality over time for the current treatment strategy (“obs”, black curve), fivefold cross-validation benchmark for the optimal DTR (“CV-DTR”, red curve) and the DTR indexed 28 (“optDTR”, blue curve), which was identified among the best performing of the considered treatment strategies. The dashed lines correspond to 95% pointwise confidence intervals

Figure 3 displays the estimated cumulative incidence function of ICU mortality under an optimal DTR (i.e. DTR with index 27 in Fig. 2 and Table 1, blue curve) as compared to that observed under current standard of care (black curve). This DTR incorporated a different threshold for pH for patients who did (pH < 7.2) vs those who did not (pH < 7.0) have oliguric KDIGO-AKI stage 2, and a threshold for potassium of 7.0 mEq/l. It is estimated to reduce 30-day ICU mortality by 2.2% [95%CI 1.3–3.8%] (from 12.7% [95%CI 11.9–13.6%] under current standard of care to 10.5% [95%CI 9.5–11.7%]) without increase in number of patients starting RRT (from 471 [95%CI 430–511] to 475 [95%CI 342–572]) as compared to current standard of care. The fivefold cross-validation benchmark for the optimal DTR resulted in 30-day ICU mortality of 10.7% (see Fig. 3), which is only slightly higher than the point estimate of 10.5% under the DTR identified as the optimal DTR on the full dataset, suggesting that the level of overoptimism was limited. For reference, Table 3 presents the cut-off values for the different absolute indications to start RRT in different large RCTs on this topic. Our analysis suggests that outcomes in these RCTs could further improve if the thresholds of the absolute indications would have been lower for pH and higher for potassium, and if a difference would have been made in the decision to start RRT between patients who did or did not reach the oliguria criterion.

**Table 3:** Criteria for in- and exclusion, absolute indications for dialysis, and disease severity in large randomized controlled trials

	AKIKI	IDEAL-ICU	ELAIN	STARRT-AKI	AKIKI 2
Eligibility criteria	KDIGO-AKI stage 3	RIFLE-F	KDIGO-AKI stage 2	KDIGO-AKI stage 2 or 3	KDGO AKI stage 3
Fluid status	Oliguria for > 72 h			Oliguria > 72 h	Oliguria > 72 h
Serum potassium	> 6 mEq/l	> 6.5 mEq/l	> 6 mEq/L	> 6.0 mEq/l	> 6.0 mEq/l
pH	< 7.15	< 7.15	–	< 7.20	< 7.15
SOFA score	10.9 ± 3.2	12.2 ± 2.9	15.6 ± 2.3	11.6 ± 3.6	11 ± 3

For serum potassium, there was no evidence to support that a potassium level threshold of 7.0 mEq/l as an absolute indication to initiate RRT in patients who have reached KDIGO-AKI stage ≥ 2 would be detrimental as compared to a lower threshold as applied in current RCTs. For pH, existing absolute criteria of pH < 7.2 seem to be adequate for oliguric patients, but for non-oliguric patients’ threshold of pH < 7.0 seems more appropriate.

## Discussion

Our results add detail to the existing literature on initiation of RRT in patients with AKI at the ICU. They suggest RRT can be delayed until serum potassium exceeds 7.0 mEq/l or persisting oliguria longer than 24–36 h is present; for pH, our results suggest the cut-off for RRT initiation depends on whether the patient is oliguric (pH < 7.2) or not (pH < 7.0). This DTR could reduce 30-day mortality and number of patients starting RRT as compared to the cut-offs proposed in current RCTs. This DTR can in a next step be evaluated in an external cohort and consecutively in an RCT. We demonstrate that decisions on the initiation of RRT for patients with AKI at an ICU can potentially be further improved by target trial emulation of RCD to assess different cut-offs for absolute indications such as pH, potassium levels, and persisting oliguria.

Several RCTs suggested that a delayed vs early start of RRT in ICU patients with AKI does not jeopardize outcome and reduces the number of patients actually starting RRT [5]. However, within these RCTs, thresholds for absolute criteria to initiate RRT without further delay are either imposed as an exclusion criterion or incorporated in delayed treatment rules. Although these absolute indications can make sense from a theoretical point of view, guidance on absolute values of their cut-offs is lacking [1, 10, 11]. We therefore evaluated different cut-offs for frequently used absolute indications to initiate RRT [3, 5–7, 10, 11]: pH, serum potassium, and persisting oligo-anuria.

Our results demonstrate that RRT can be delayed until specific thresholds for these criteria are met. They suggest that higher thresholds of potassium (up to > 7.0 mEq/l) can be applied as absolute criterion to initiate RRT. For pH, the optimal cut-off depends on whether or not the patient also meets the KDIGO-AKI stage 2 oliguria criterion (pH < 7.2) or not (pH < 7.0). Our results suggest that RRT is not without risks and that increased mortality may ensue when patients are started on RRT without appropriate indication. Persisting oliguria for 24–36 h after reaching AKI stage ≥ 2 was also a criterion to start RRT, which is in line with the results of the recent AKIKI2 trial [6] and also with the evidence underpinning the important negative impact of fluid overload in patients with AKI [28]. Particularly persistent volume loading not resulting in restoring urine output seems to be a strong negative predictor, and in such cases starting RRT seems to be preferable to further attempts to fluid load the patient [29, 30]. Our algorithm takes persisting oliguria as a decision parameter rather than other parameters of fluid status, such as fluid balance, blood pressure, or oedema, which in patients at ICU all have a low diagnostic value [29]. Nevertheless, and of importance, the standardized operating protocol in our ICU specifies the use of a fluid challenge [30] in patients with impending oliguria when hypovolaemia is suspected.

Our approach follows the concept that timing of RRT should not be phrased in terms of “early vs late” but rather in terms of specific criteria [31] to start or not. Existing RCTs indeed differ on the point at which patients become eligible for inclusion, with some using KDIGO-AKI stage ≥ 2, and others stage 3, and in thresholds for criteria considered absolute indications. Also, operationalization of the KDIGO-AKI criteria themselves might already differ between studies and centres, leading to substantial differences in which patients will or will not be included in the study, and at which timepoint [24, 32]. The present paper illustrates how RCD can be leveraged to inform decisions about RRT initiation, and how more optimal cut-offs for absolute indications can be estimated. The use of RCD in this setting offers some advantages over an RCT. It allowed us to include all patients from the moment they fulfilled the criteria of KDIGO-AKI stage ≥ 2, which makes our results likely more generalizable to patients who fulfil those criteria in a typical ICU. This contrasts with results of an RCT, where external validity is often problematic due to very strict in- and exclusion criteria [33–36]. Even so, however, some caution is needed as there might still be unidentified differences in case mix even between ICUs.

Our approach not only yields broader representativeness, but also suggests randomization would be acceptable in future RCTs comparing timing strategies for RRT initiation at potassium levels of > 7.0 mEql or pH levels of < 7.0 (non-oliguric) or < 7.2 (oliguric patients).

Further signs of the broader generalizability to a general ICU patient population as compared to those of recent RCTs are the relatively low percentage of patients in our cohort initiating RRT (around 8.4%) and the relatively low mortality (around 12.7%). Although deviating substantially from those reported in RCTs on this topic [5], they are in line with what is expected for patients with KDIGO-AKI stage ≥ 2 in a modern ICU [24, 37].

DTRs that initiated RRT at potassium levels < 7.0 mEq/l were estimated not to improve outcome and resulted in a substantial increase in the number of patients initiating RRT. Similar observations have been made in other RCTs in which high serum potassium was an absolute indication [5]. For pH, the optimal threshold depends on whether or not patients are oliguric. In non-oliguric patients, DTRs with higher pH thresholds (pH < 7.2 rather than pH < 7.0) as criterion for initiating RRT resulted in an increase in the number or patients initiating RRT, and a substantial increase in mortality. In contrast, in oliguric patients, a higher vs a lower (7.2 vs 7.0) threshold for pH appears to be associated with lower 30-day ICU mortality. Such a distinction is not made in any of the existing RCTs. Likely, RRT is not very useful in patients with isolated acidosis, as this is often associated with severe other non-renal conditions that cannot be reversed by RRT [38], whereas if the (less expressed) acidosis is mainly related to kidney failure and oliguria, this is more likely to be modifiable by RRT [38].

The strengths of this study are the large data set with few missing data (see Additional file 1: Appendix C) and the use of state-of-the-art statistical causal inference methodology. Another strength is that we include a wide patient mix, which makes that results can be generalized to other settings. However, this heterogeneity makes that in specific subpopulations, other DTRs might be more optimal than the one identified to be most optimal in the overall population. Some other limitations need to be indicated. The validity of conclusions from RCD relies on a set of causal assumptions. We assumed no unmeasured confounding, which requires that all factors prognostic for the outcome that are considered by clinicians when deciding on the RRT initiation have been properly adjusted for. We consider the assumption of no unmeasured confounding to be justifiable in our study since ICU patients remain in a highly controlled environment and many patient characteristics are being measured and monitored. This allowed us to include a broad set of potential confounders (both baseline and time-varying). Because residual confounding can never be excluded in observational studies, the optimal DTR as identified in our study should ideally be validated in a well-designed and well-conducted RCT. Also, in our analysis we have assumed no interference, i.e. decisions about RRT initiation for a particular patient do not impact treatment decisions (or their timing) for other patients, e.g. due to scarceness of resources. We consider this to be justifiable as access to RRT was never a limitation in our centre. Further, we report the performance of the selected DTR on the same data set used to choose it from the set of considered DTRs, which may have led to overoptimism in the reported performance. To assess the degree of overoptimism, we performed a fivefold cross-validation for the optimal DTR [26] as a form of internal validation of our results and to assess the expected performance of the optimal DTR on a new dataset. This fivefold cross-validation benchmark for the optimal DTR resulted in 30-day ICU mortality of 10.7%, demonstrating that the extent of overoptimism of performance was in our analysis only very limited [26].

Further, the thresholds used in our study are values in patients who already received maximal conservative (non-RRT) measures to control pH, potassium, and/or achieve adequate diuresis, as stipulated in the standardized operating protocols in our ICU. The clinical question we intended to address our target trial emulation reported in our paper is thus “in a patient with criterion *X* (e.g. persisting oliguria) despite maximal non-RRT treatment (e.g. diuretics) is it better to postpone RRT initiation or start immediately”. It is important to note that another relevant clinical question such as “is it better to immediately start RRT or start diuretics in oliguric patients with AKI stage 2” cannot be answered by a target trial emulation based on our current dataset, simply because there would be no or only ample reference cases where no diuretics would have been tried before RRT was considered [39].

Last, we conducted our observational analysis within a target trial emulation framework to generate hypotheses about optimal treatment strategies, which should ideally be validated in an RCT [14]. As a consequence, our results should also be tested in a large multicentric RCT.

In conclusion, our results provide potential thresholds for the absolute criteria to start RRT. They indicate that in patients with KDIGO-AKI stage ≥ 2, initiation of RRT could be postponed until serum potassium > 7.0 mEq/l, or persisting or worsening oliguria as indicated by the oliguria criterion of KDIGO-AKI stage 3, whereas for pH, the threshold is 7.2 for oliguric and 7.0 for non-oliguric patients. As target trials based on observational data are hypothesis generating, the proposed DTR should be validated in an RCT.

## Supplementary Information

**Additional file 1. Appendix part A:** Additional plots illustrating 30-day ICU mortality for different considered DTRs. **Appendix part B:** Simplified example illustrating cloning-censoring-weighting approach. **Appendix part C:** Overview of the variables used in the analysis and percentage of missing values at the baseline. **Appendix part D:** Propensity score model. **Appendix part E:** Inverse-probability-of-censoring weights.

## Data Availability

According to GDPR rules, and in line with the informed consent and the approval of the ethics committee of the University Hospital of Ghent, full, non-aggregated data can only be made available by the authors after permission of the ethics committee. Interested parties can address the authors to discuss an eventual project proposal to be submitted to the ethics committee.
