# Supplementary material for: Timing of dialysis in acute kidney injury using routinely collected data and dynamic treatment regimes
Source: Crit Care. 2022 Nov 28;26:365. doi: 10.1186/s13054-022-04252-1 (PMC9706864; doi:10.1186/s13054-022-04252-1)
Supplement: Supplementary file 1 — Additional file 1. Appendix part A: Additional plots illustrating 30-day ICU mortality for different considered DTRs. Appendix part B: Simplified example illustrating cloning-censoring-weighting approach. Appendix part C: Overview of the variables used in the analysis and percentage of missing values at the baseline. Appendix part D: Propensity score model. Appendix part E: Inverse-probability-of-censoring weights. [file 13054_2022_4252_MOESM1_ESM.docx]

**Appendix**

1. **Additional plots illustrating 30-day ICU mortality for different considered DTRs**

**Appendix Figure 1: 30-day ICU mortality for different DTRs**

**
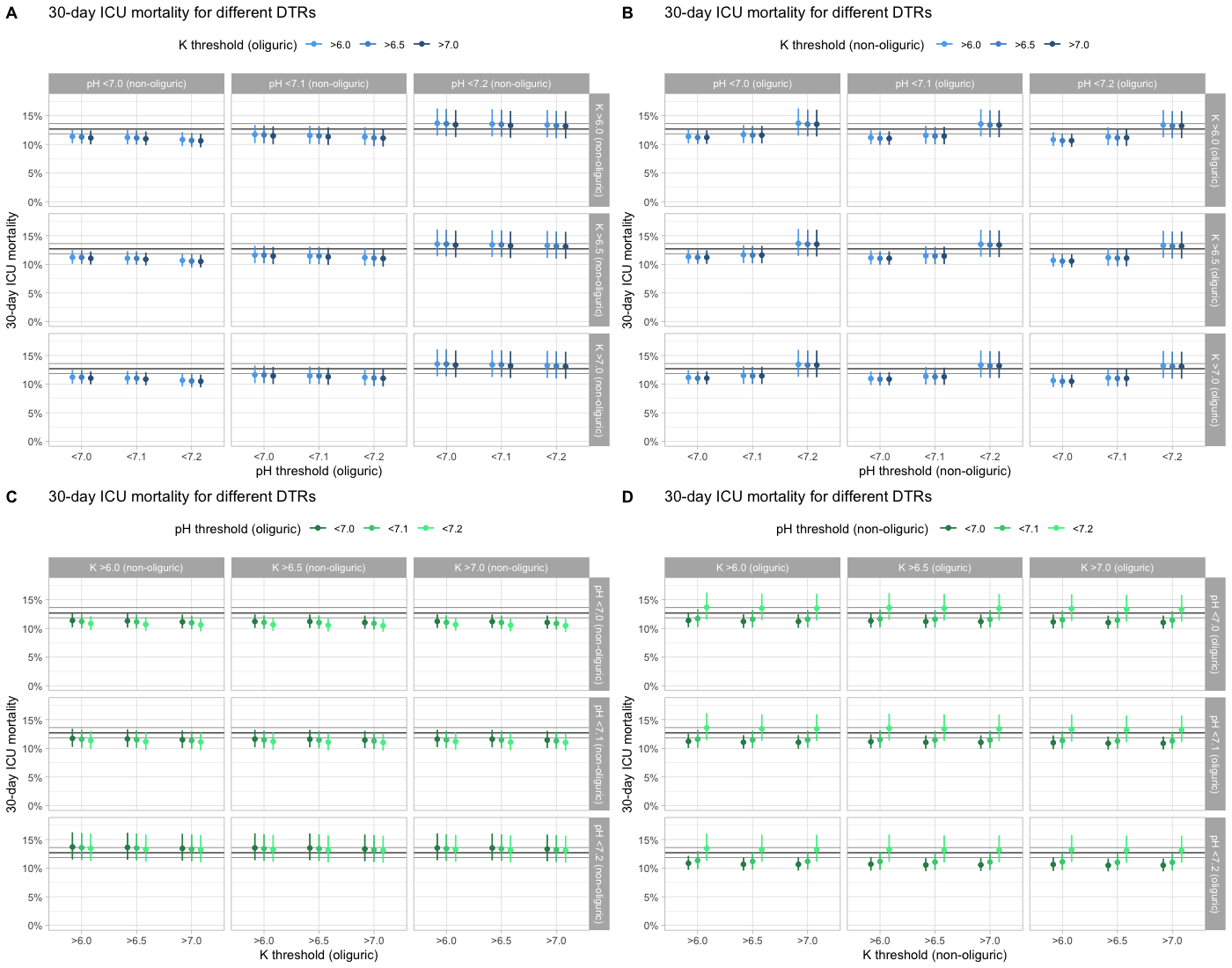
**

1. **Simplified example illustrating cloning-censoring-weighting approach**

As an example, consider a data set containing information on two patients observed over 5 days. Suppose we want to compare two dynamic treatment regimes "start RRT in AKI–diagnosed patients if pH fell below 7.1 in the last 24h" and "start RRT in AKI–diagnosed patients if pH fell below 7.2 in the last 24h". We therefore make two copies of the original data set – one for each treatment regime that we consider – and stack them on top of each other to obtain an extended data set (see Appendix Table 1). The compatibility indicator in Appendix Table 1 corresponds to the compatibility status of the patient at the end of the day with value 1 denoting being compatible with considered DTR at a given timepoint and value 0 corresponding to no longer being compatible with that DTR. Patient is compatible with particular DTR at a time k, if he/she has not been artificially censored for this DTR up to that timepoint. Given that a patient is compatible with a considered treatment strategy at time k, we can determine the compatibility indicator on the following day by checking whether the treatment decision based on the considered treatment strategy coincides with the observed treatment. For the DTR "start RRT in AKI–diagnosed patients if pH fell below x in the last 24h" a given patient would remain compatible if the minimal level of pH in the last 24h fell below the threshold x and the treatment has been initiated or if the minimal level of pH did not fall below the threshold x in the last 24h and treatment has not been started. Once the patient ceases to be compatible with a particular treatment regime at some time point, then he/she will be incompatible (i.e. artificially censored) with this regime for all future time points. The patients who are compatible with a particular DTR at the time of experiencing one of the events – ICU death or ICU discharge – remain compatible with the considered dynamic treatment regime until the end of the study. Furthermore, to address the selection bias due to artificial censoring of patients who cease to be compatible with considered DTR and to address the issue of competing risks, with ICU mortality being the outcome of interest and ICU discharge being the competing event, we have used an inverse-probability-of-censoring-weighted Aalen-Johansen estimator^1^.

**Appendix Table 1: Simplified example illustrating cloning-censoring-weighting approach for evaluation of DTRs.**

| **ID** | **Time (Days)** | **DTR** | **Observed Treatment** | **Min. pH (last 24h)** | **Compatibility Indicator** |
| --- | --- | --- | --- | --- | --- |
| **1** | 1 | 7.1 | 0 | 7.29 | 1 |
| **1** | 2 | 7.1 | 0 | 7.24 | 1 |
| **1** | 3 | 7.1 | 1 | 7.08 | 1 |
| **1** | 4 | 7.1 | 1 | 7.29 | 1 |
| **1** | 5 | 7.1 | 1 | 7.29 | 1 |
| **2** | 1 | 7.1 | 0 | 7.3 | 1 |
| **2** | 2 | 7.1 | 0 | 7.29 | 1 |
| **2** | 3 | 7.1 | 1 | 7.19 | 0 |
| **2** | 4 | 7.1 | 1 | 7.32 | 0 |
| **2** | 5 | 7.1 | 1 | 7.32 | 0 |
| **1** | 1 | 7.2 | 0 | 7.29 | 1 |
| **1** | 2 | 7.2 | 0 | 7.24 | 1 |
| **1** | 3 | 7.2 | 1 | 7.08 | 1 |
| **1** | 4 | 7.2 | 1 | 7.29 | 1 |
| **1** | 5 | 7.2 | 1 | 7.29 | 1 |
| **2** | 1 | 7.2 | 0 | 7.3 | 1 |
| **2** | 2 | 7.2 | 0 | 7.29 | 1 |
| **2** | 3 | 7.2 | 1 | 7.19 | 1 |
| **2** | 4 | 7.2 | 1 | 7.32 | 1 |
| **2** | 5 | 7.2 | 1 | 7.32 | 1 |

1. **Overview of the variables used in the analysis and percentage of missing values at the baseline.**

Missingness indicator

Tables below present the percentage of missing values at the baseline for different variables. For each variable that had missing values we have constructed an indicator of missingness (“NA indicator”), which has been set to 1, if the value was missing, and 0 otherwise.

Imputation of missing values

For time-varying covariates we have applied “last observation carried forward”, whenever there was a missing value at a later (than baseline) timepoint, and earlier observations were available. This corresponds to adjusting for the last recorded measurement. Whenever there was a missing value at the baseline or no earlier observations were available, we have imputed 0. This is because we use a missing indicator method, which includes an indicator of whether the value is missing or not, as described above.

Impact of missing values on considered DTRs

It is important to note that the considered DTRs were designed not to initiate RRT based on missing values (or values imputed in place of missing values). For example, it could happen that a patient has a missing value of pH at time 0, hence value 0 would be imputed as described above. Now, potentially this could lead to RRT initiation in these patients through the component of considered DTRs “initiate RRT if pH fell below threshold x”. We have prevented such situations through requiring in implemented DTRs that only observed values of pH, serum potassium and fluid output could be used to evaluate DTR at given timepoint, which is analogous to clinical setting where clinicians take a decision about treatment initiation only based on information available up to that timepoint. That is, in case of a patient whose value of pH is missing at time 0 (however the values serum potassium and fluid output were measured) only potassium and oliguric component of DTR could lead to RRT initiation at time 0 (and until first pH measurement has been recorded).

**Baseline Covariates**

| **Variable** | **% of Missingness** |
| --- | --- |
| Year of admission | 0% |
| Admission time (“Day”, “Night”) | 0% |
| Admission category ("No surgery”, “Planned surgery”, “Emergency surgery”, “No Answer”) | 0.7% |
| Gender (“F”, “M”) | 0% |
| Age on admission | 0% |
| Indicator of chronic renal failure at the time of ICU admission | 0% |
| Weight | 0% |
| Time from ICU admission until KDIGO-AKI stage≥2 diagnosis (in hours) | 0% |

**Time-varying Covariates**

| **Variable** | **% of Missingness**  **(at the baseline)** |
| --- | --- |
| Oliguric KDIGO-AKI stage 2 indicator | 0% |
| Oliguric KDIGO-AKI stage 3 indicator | 0% |
| Creatinine KDIGO-AKI stage 2 indicator | 0% |
| Creatinine KDIGO-AKI stage 3 indicator | 0% |
| Indicator for oliguria (defined as urine output <0.3ml/kg/h for 48h) | 92.1%^^[[1]](#footnote-1)^^ |
| Indicator for oliguria (defined as urine output <0.5ml/kg/h for 48h) | 92.1%^^[[2]](#footnote-2)^^ |
| Fraction of inspired oxygen (FiO_2_) | 17.3% |
| Serum potassium [in mEq/l]] | 4.4% |
| Serum magnesium [in mEq/l]] | 4.5% |
| Partial pressure of oxygen (PaO_2_) [in mmHg] | 10.5% |
| Ratio of arterial oxygen concentration to the fraction of inspired oxygen (P/F ratio) | 25.3% |
| Arterial pH value | 10.5% |
| Serum urea [in mg/dL] | 4.5% |
| Arterial oxygen saturation (SpO_2_) [%] | 2.7% |
| Cumulative fluid intake [in mL] | 0.02% |
| Cumulative fluid output [in mL] | 0.02% |
| SOFA cardio score | 2.8% |
| SOFA CNS score | 2.8% |
| SOFA coagulation score | 2.8% |
| SOFA cardio score | 2.8% |
| SOFA respiration score | 2.8% |
| SOFA renal score | 2.8% |
| Septic shock indicator | 0% |
| Severe sepsis indicator | 0% |
| Do Not Resuscitate (DNR) code | 0% |
| Dialysis restriction indicator | 0% |
| RRT initiation indicator | 0% |

**Study outcomes**

| ICU death indicator | 0% |
| --- | --- |
| ICU discharge indicator | 0% |

1. **Propensity score model**

To apply inverse-probability-weighting technique in our analysis we have fitted a propensity score model, i.e. a model predicting probability of receiving RRT for a particular patient. Since we are interested in RRT initiation, hence we have restricted fitting the propensity score model to patients, who have not received RRT yet, who had no dialysis restrictions and whose DNR code is below 3 at the time of treatment initiation decision point.

Model choice

We estimated propensity score using a pooled logistic regression generalized additive model (GAM) including main effects of the above-mentioned baseline and time-varying covariates and time (i.e. “Time since AKI stage 2 diagnosis (in days)). Time has been modelled using splines with 10 knots.

Confounding adjustment

The main objective when building a propensity score model was confounding adjustment, therefore we have included in the propensity score model all factors prognostic for the outcome that are considered by clinicians when deciding on the RRT initiation. In our analysis we consider a setting in which decisions on RRT initiation for each patient are made every 24h. It may happen that a patient have multiple measurements of a particular variable during 24h. We aggregate this information into a single value by taking the highest value in the last 24h (e.g. for FiO_2_, serum potassium, serum magnesium, serum urea, SOFA subscores), the lowest value in the last 24h (e.g. for PaO_2_, P/F ratio, pH, SpO_2_), the most recent value (e.g. for DNR code) or a cumulative value in the last 24h (e.g. for fluid intake per kg, fluid output per kg). Furthermore, for each time-varying covariate additionally to the value from the most recent 24h period we include at each decision point also the value from the previous 24h period (i.e. LAG1 variable) to be able to capture change in the values of covariates over time in our model.

Variable selection

We did not apply variable selection procedures for the propensity score model to prevent bias in estimates and standard errors that would otherwise arise due to variable selection uncertainty.

**Appendix Table 2: Coefficients of the propensity score model (on odds-ratio scale, 95% confidence intervals and p-values).**

| Variable | OR^1^ | 95% CI^1^ | p-value |
| --- | --- | --- | --- |
| Year of admission |  |  |  |
| 2013 | — | — |  |
| 2014 | 0.76 | 0.52, 1.10 | 0.14 |
| 2015 | 0.82 | 0.55, 1.20 | 0.3 |
| 2016 | 0.62 | 0.41, 0.92 | 0.019 |
| 2017 | 0.63 | 0.42, 0.94 | 0.023 |
| Time of admission |  |  |  |
| Day | — | — |  |
| night | 0.94 | 0.75, 1.17 | 0.6 |
| Admission category |  |  |  |
| No surgery | — | — |  |
| Planned surgery | 0.65 | 0.48, 0.87 | 0.003 |
| Emergency surgery | 1.28 | 0.92, 1.78 | 0.15 |
| No Answer | 0.18 | 0.02, 1.65 | 0.13 |
| Gender |  |  |  |
| F | — | — |  |
| M | 0.80 | 0.63, 1.01 | 0.061 |
| Age on admission | 0.99 | 0.98, 1.00 | 0.052 |
| Chronic renal failure |  |  |  |
| FALSE | — | — |  |
| TRUE | 1.04 | 0.82, 1.33 | 0.7 |
| Weight | 0.99 | 0.99, 1.00 | 0.020 |
| Time from ICU admission until AKI2 diagnosis  (In hours) | 1.00 | 1.00, 1.01 | 0.10 |
| Oliguric AKI stage 2 | 3.17 | 1.85, 5.42 | <0.001 |
| Oliguric AKI stage 2 LAG1 | 1.05 | 0.62, 1.79 | 0.9 |
| Oliguric AKI stage 3 | 2.93 | 1.98, 4.34 | <0.001 |
| Oliguric AKI stage 3 LAG1 | 0.57 | 0.34, 0.94 | 0.029 |
| Creatinine AKI stage 2 | 5.51 | 3.08, 9.84 | <0.001 |
| Creatinine AKI stage 2 LAG1 | 0.73 | 0.41, 1.28 | 0.3 |
| Creatinine AKI stage 3 | 2.13 | 1.55, 2.92 | <0.001 |
| Creatinine AKI stage 3 LAG1 | 0.56 | 0.37, 0.85 | 0.007 |
| Persisting oliguria (48h) | 1.61 | 1.00, 2.60 | 0.048 |
| Persisting oliguria (48h) – NA ind. | 0.99 | 0.61, 1.60 | >0.9 |
| Persisting oliguria (48h) LAG1 | 0.72 | 0.37, 1.40 | 0.3 |
| Persistent oliguria (48h) LAG1 – NA ind. | 1.51 | 0.94, 2.42 | 0.092 |
| Max. FiO2 (last 24h) | 1.00 | 1.00, 1.00 | 0.7 |
| Max. FiO2 (last 24h) – NA ind. | 1.12 | 0.67, 1.87 | 0.7 |
| Max. FiO2 (last 24h) LAG1 | 1.00 | 1.00, 1.00 | 0.7 |
| Max. FiO2 (last 24h) LAG1 – NA ind. | 1.55 | 0.77, 3.15 | 0.2 |
| Max. K (last 24h) | 1.56 | 1.32, 1.84 | <0.001 |
| Max. K (last 24h) - NA ind. | 11.4 | 0.04, 3,143 | 0.4 |
| Max. K (last 24h) LAG1 | 0.98 | 0.77, 1.24 | 0.9 |
| Max. K (last 24h) LAG1 - NA ind. | 0.65 | 0.00, 434 | 0.9 |
| Max. Mg (last 24h) | 1.73 | 1.12, 2.68 | 0.014 |
| Max. Mg (last 24h) - NA ind. | 0.48 | 0.00, 61.0 | 0.8 |
| Max. Mg (last 24h) LAG1 | 1.34 | 0.79, 2.28 | 0.3 |
| Max. Mg (last 24h) LAG1 - NA ind. | 0.39 | 0.00, 126 | 0.7 |
| Min. PaO2 (last 24h) | 1.00 | 1.00, 1.00 | 0.6 |
| Min. PaO2 (last 24h) - NA ind. | 3.42 | 0.00, 57,396 | 0.8 |
| Min. PaO2 (last 24h) LAG1 | 1.00 | 1.00, 1.01 | 0.3 |
| Min. PaO2 (last 24h) LAG1 - NA ind. | 0.11 | 0.00, 112,889 | 0.8 |
| Min. P/F ratio (last 24h) | 1.00 | 1.00, 1.00 | 0.3 |
| Min. P/F ratio (last 24h) - NA ind. | 0.95 | 0.52, 1.70 | 0.9 |
| Min. P/F ratio (last 24h) LAG1 | 1.00 | 1.00, 1.00 | 0.6 |
| Min. P/F ratio (last 24h) LAG1 - NA ind. | 0.61 | 0.27, 1.36 | 0.2 |
| Min. pH (last 24h) | 0.25 | 0.08, 0.79 | 0.019 |
| Min. pH (last 24h) - NA ind. | 0.00 | 0.00, 7.02 | 0.10 |
| Min. pH (last 24h) LAG1 | 1.07 | 0.77, 1.47 | 0.7 |
| Min. pH (last 24h) LAG1 - NA ind. | 13.3 | 0.00, 16,031,817 | 0.7 |
| Max. urea (last 24h) | 1.01 | 1.00, 1.01 | <0.001 |
| Max. urea (last 24h) - NA ind. | 0.74 | 0.03, 16.6 | 0.8 |
| Max. urea (last 24h) LAG1 | 1.00 | 1.00, 1.01 | 0.3 |
| Max. urea (last 24h) LAG1 - NA ind. | 0.37 | 0.01, 11.5 | 0.6 |
| Min. SpO2 (last 24h) | 1.00 | 0.99, 1.01 | 0.7 |
| Min. SpO2 (last 24h) - NA ind. | 1.77 | 0.58, 5.41 | 0.3 |
| Min. SpO2 (last 24h) LAG1 | 1.01 | 0.99, 1.02 | 0.3 |
| Min. SpO2 (last 24h) LAG1 - NA ind. | 1.04 | 0.16, 6.79 | >0.9 |
| Fluid intake per kg (last 24h) | 1.00 | 1.00, 1.00 | >0.9 |
| Fluid intake per kg (last 24h) - NA ind. | 0.00 | 0.00, Inf | >0.9 |
| Fluid intake per kg (last 24h) LAG1 | 1.00 | 1.00, 1.00 | 0.9 |
| Fluid intake per kg (last 24h) LAG1 - NA ind. | 1.00 | 1.00, 1.00 |  |
| Fluid output per kg (last 24h) | 0.99 | 0.99, 1.00 | <0.001 |
| Fluid output per kg (last 24h) - NA ind. | 1.00 | 1.00, 1.00 |  |
| Fluid output per kg (last 24h) LAG1 | 1.01 | 1.00, 1.01 | 0.003 |
| Fluid output per kg (last 24h) LAG1 - NA ind. | 86.6 | 5.53, 1,355 | 0.001 |
| Max. SOFA cardio (last 24h) | 1.51 | 1.37, 1.67 | <0.001 |
| Max. SOFA cardio (last 24h) - NA ind. | 1.00 | 1.00, 1.00 |  |
| Max. SOFA cardio (last 24h) LAG1 | 0.80 | 0.71, 0.90 | <0.001 |
| Max. SOFA cardio (last 24h) LAG1 - NA ind. | 1.00 | 1.00, 1.00 |  |
| Max. SOFA CNS (last 24h) | 0.96 | 0.88, 1.04 | 0.3 |
| Max. SOFA CNS (last 24h) - NA ind. | 1.00 | 1.00, 1.00 |  |
| Max. SOFA CNS (last 24h) LAG1 | 1.10 | 0.99, 1.23 | 0.077 |
| Max. SOFA CNS (last 24h) LAG1 - NA ind. | 1.00 | 1.00, 1.00 |  |
| Max. SOFA coagulation (last 24h) | 1.26 | 1.12, 1.42 | <0.001 |
| Max. SOFA coagulation (last 24h) - NA ind. | 0.93 | 0.41, 2.09 | 0.9 |
| Max. SOFA coagulation (last 24h) LAG1 | 0.92 | 0.77, 1.09 | 0.3 |
| Max. SOFA coagulation (last 24h) LAG1 - NA ind. | 1.00 | 1.00, 1.00 |  |
| Max. SOFA liver (last 24h) | 1.28 | 1.12, 1.45 | <0.001 |
| Max. SOFA liver (last 24h) - NA ind. | 1.00 | 1.00, 1.00 |  |
| Max. SOFA liver (last 24h) LAG1 | 0.96 | 0.81, 1.15 | 0.7 |
| Max. SOFA liver (last 24h) LAG1 - NA ind. | 0.68 | 0.20, 2.31 | 0.5 |
| Max. SOFA respiration (last 24h) | 1.24 | 1.09, 1.43 | 0.002 |
| Max. SOFA respiration (last 24h) - NA ind. | 1.00 | 1.00, 1.00 |  |
| Max. SOFA respiration (last 24h) LAG1 | 1.03 | 0.86, 1.23 | 0.8 |
| Max. SOFA respiration (last 24h) LAG1 - NA ind. | 1.00 | 1.00, 1.00 |  |
| Max. SOFA renal (last 24h) | 1.67 | 1.48, 1.89 | <0.001 |
| Max. SOFA renal (last 24h) - NA ind. | 1.00 | 1.00, 1.00 |  |
| Max. SOFA renal (last 24h) LAG1 | 1.09 | 0.95, 1.26 | 0.2 |
| Max. SOFA renal (last 24h) LAG1 - NA ind. | 1.00 | 1.00, 1.00 |  |
| Septic shock | 1.15 | 0.65, 2.05 | 0.6 |
| Septic shock LAG1 | 0.73 | 0.32, 1.64 | 0.4 |
| Severe sepsis | 1.48 | 0.43, 5.12 | 0.5 |
| Severe sepsis LAG1 | 2.18 | 0.46, 10.4 | 0.3 |
| Last DNR code |  |  |  |
| 0 | — | — |  |
| 1 | 0.09 | 0.01, 0.64 | 0.016 |
| 2 | 0.92 | 0.30, 2.82 | 0.9 |
| Last DNR code LAG1 |  |  |  |
| 0 | — | — |  |
| 1 | 1.65 | 0.19, 14.3 | 0.7 |
| 2 | 0.29 | 0.03, 3.31 | 0.3 |
| s (Time since AKI stage 2 diagnosis (in days)) |  |  | 0.6 |
| ^1^OR = Odds Ratio, CI = Confidence Interval | | | |

1. **Inverse-probability-of-censoring weights**

Following the methodology described in Morzywołek et al^1^. (2022), we have computed inverse-probability-of-censoring weights using the propensity scores obtained from the propensity score model described above.

Trimming of the weights

To avoid overly high impact of a single individual on the results of our analysis we have decided to trim the obtained inverse-probability-of-censoring-weights at level of 30. This impacted inverse-probability-of-censoring weights of 6 individuals.

**Appendix Figure 2: Inverse-probability of censoring weights**


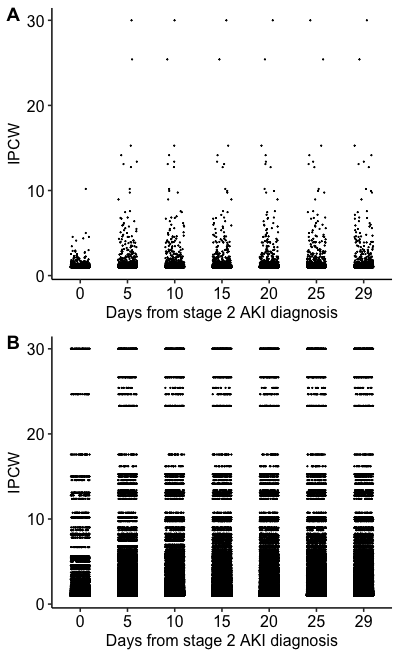


Panel A presents inverse-probability-of-censoring weights (trimmed at level 30) at selected timepoints used to evaluate DTR with indexed 28 (see Table 3 in the main text). Panel B presents inverse-probability-of-censoring weights (trimmed at level 30) at selected timepoints used to evaluate all of the 81 considered DTRs.

1 Morzywolek, P. On Estimation and Cross-validation of Dynamic Treatment Regimes with Competing Risks. *Statistics in medicine* **in press** (2022).

1. High missingness percentage related to the fact that patient was under observation less than 48h. [↑](#footnote-ref-1)
2. High missingness percentage related to the fact that patient was under observation less than 48h. [↑](#footnote-ref-2)
